# A Case of Primary Epstein-Barr Virus Infection Masquerading As Drug Reaction With Eosinophilia and Systemic Symptoms

**DOI:** 10.7759/cureus.33782

**Published:** 2023-01-15

**Authors:** Laura J O'Keefe, Kathryn M Burtson

**Affiliations:** 1 Internal Medicine, Brooke Army Medical Center, San Antonio, USA; 2 Internal Medicine, Wright-Patterson Air Force Base Medical Center, Dayton, USA

**Keywords:** ebv-associated hepatitis, cryptic hepatitis, ebv hepatitis, hepatitis, antibiotic reaction, macular rash, ebv, dress

## Abstract

In this case report, we discuss the diagnostic dilemma presented by a patient admitted for elevated liver enzymes and rash, who had a history of recent amoxicillin use. This presentation initially appeared to fit the criteria for Drug Reaction with Eosinophilia and Systemic Symptoms (DRESS) syndrome. However, histologic evaluation determined the rash was consistent with Miliaria rubra rather than the lymphocytic infiltrate of DRESS. This necessitated broad diagnostic testing to determine the underlying etiology of the patient’s syndrome. Serology subsequently demonstrated primary Epstein-Barr Virus (EBV) infection, which explained her acute liver injury. Her eosinophilia was potentially related to an allergic reaction to surgical tape but was never definitely diagnosed. This case demonstrates the importance of maintaining a wide differential even when clinical diagnostic criteria are apparently met.

## Introduction

Drug Reaction with Eosinophilia and Systemic Symptoms (DRESS) is a life-threatening drug-induced hypersensitivity syndrome characterized by a morbilliform eruption, marked eosinophilia greater than 700 cells per microliter, and end-organ dysfunction. Drugs that are known to cause DRESS range from over-the-counter drugs such as non-steroidal anti-inflammatory drugs (NSAIDs), to antiepileptic drugs such as carbamazepine, and, notably, beta-lactam antibiotics such as amoxicillin. The annual incidence of DRESS is approximately 1-2 per 100,000 patients [1].

The Epstein-Barr virus (EBV) is a near-ubiquitous virus best known as the cause of infectious mononucleosis in adolescents [2]. This typically presents with nonspecific symptoms of fever and malaise, followed by cervical lymphadenopathy and pharyngitis. The manifestations of EBV in older adults are less well-established, but several case reports describe a syndrome of acute hepatitis [3-5]. Many infections are subclinical; therefore, the incidence is poorly estimated. However, the prevalence of EBV infection, based on EBV antibody positivity, reaches 90 to 95% by adulthood. This report presents an unusual case of a patient with hepatocellular injury, peripheral eosinophilia, and recent amoxicillin use. Despite initial concern for DRESS, the patient was found to have a primary EBV infection.

This article was previously presented as an ePoster at the 2021 American College of Physicians (ACP) National Abstract Competition on May 14, 2021.

## Case presentation

The patient was a 64-year-old woman who presented to this institution with worsening rash, nausea, and headache two weeks after beginning a course of amoxicillin. She was found to have a hepatocellular injury, peripheral eosinophilia, and a fine macular rash on the back, chest, and arms. Three weeks prior to this admission, the patient underwent left total knee replacement and subsequently developed a pruritic rash around her surgical scar. She was directed to take amoxicillin by her surgeon to treat the rash and subsequently noticed a different rash on her back, chest, and arms. She presented to the Emergency Department (ED) for evaluation of both rashes about one week later. The rash around her surgical scar was attributed to contact dermatitis from the surgical tape, whereas the rash on her trunk was attributed to a drug reaction from amoxicillin. She was given dexamethasone for the rash on her trunk and instructed to discontinue amoxicillin.

Over the following week, the patient developed nausea, hypertension, headache, and persistent rash, so she returned to the ED. She was found to have anaspartate aminotransferase (AST) of 400 and an alanine aminotransferase (ALT) greater than 500 as well as 12.6% peripheral eosinophils. These findings prompted concern for DRESS syndrome in light of her recent amoxicillin use. The patient was admitted to the internal medicine service. She denied right upper quadrant tenderness and endorsed only intermittent nausea and headache. On physical exam, she had a faint, macular, mildly pruritic rash across her back, shoulders, and chest which remained stable. Her liver-associated enzymes continued to rise during her inpatient admission (Table 1).

**Table 1:** Liver-associated enzyme results AST: aspartate aminotransferase; ALT: alanine aminotransferase; Alk Phos: alkaline phosphatase; T-Bili: bilirubin test; GGT: gamma-glutamyl transferase

Date	AST (8-48 U/L)	ALT (7-55 U/L)	Alk Phos (40-129 U/L)	T-Bili (0.1-1.2 mg/dL)	GGT (8-61 U/L)
22-Jun	400.2 U/L	>500 U/L	524 U/L	3.8 mg/dL	686 U/L
23-Jun	409.2 U/L	>500 U/L	526 U/L	4.5 mg/dL	
24-Jun	436.9 U/L	>500 U/L	552 U/L	5.9 mg/dL	
27-Jun	343.0 U/L	>500 U/L	761 U/L	8.5 mg/dL	
30-Jun	141.5 U/L	342 U/L	717 U/L	4.1 mg/dL	1265 U/L

DRESS syndrome was highest on the initial differential, which also included drug rash and other causes of hepatitis. The benign appearance of the rash on the exam prompted a dermatology consult. Biopsy of the rash demonstrated findings consistent with miliaria rubra, rather than the inflammatory pattern rich with cutaneous effector lymphocytes which would normally characterize the rash of DRESS [6]. Viral hepatitis serologies were negative, with normal values for ceruloplasmin, ferritin, iron, and antinuclear antibodies (ANA) (Table 2).

**Table 2:** Ancillary testing EBV: Epstein-Barr Virus; HHV-6: human herpesvirus 6; ANA: antinuclear antibody; IgM: immunoglobulin M; IgG: immunoglobulin G; CK: creatine kinase

EBV Panel	
EBV capsid IgM	detected
EBV capsid IgG	detected
EBV nuclear Ab IgG	detected
CMV Panel	
IgM	not detected
IgG	not detected
Viral Hepatitis	
Chronic hepatitis panel	not detected
HHV-6	not detected
Autoimmune Panel	
Nuclear Ab Qual	not detected
Mitochondrial Ab	not detected
Smooth muscle Ab	not detected
Liver-kidney microsomal Ab	1.3 (0.0-20.0)
ANA	not detected
Immunoglobulin Panel	
IgA	373 (87-352)
IgG	741 (586-1602)
IgM	76 (26-217)
Latex Ab IgE	<0.35
Miscellaneous	
CK	55 (30-223)
Acetaminophen	5.4 (10-30)
Ceruloplasmin	32 (16-45)
Iron	114 (50-212)
Transferrin	245 (200-360)

The patient’s liver function stabilized, and she was discharged with close follow-up. After discharge, EBV serology returned positive for primary infection. The patient was monitored as an outpatient and her liver function returned to normal within a few months.

## Discussion

The diagnosis of DRESS requires the presence of an offending drug, a rash, elevated eosinophils, and organ system involvement. DRESS can be particularly difficult to recognize given the variety of manifestations the organ system involvement can take, and the variety of drugs that can be implicated. Unlike some other drug reactions, there is no association with a human leukocyte antigen (HLA) allele or other signifier of genetic susceptibility that could improve the ease of diagnosis or provide a definitive diagnosis. Liver function abnormalities can occur in up to 70% of patients in the acute phase of DRESS, but this is neither sensitive nor specific. Reactivation of viruses such as EBV and cytomegalovirus (CMV) can occur following the onset of DRESS [7]. However, others postulate that the reactivation of viruses may precede and lead to DRESS. This is further supported by evidence of EBV-positive T-lymphocytes present in organs affected by DRESS in symptomatic patients [8]. Even outside of the context of DRESS, EBV can cause hepatic injury and lead to elevations in liver function tests or even trigger autoimmune hepatitis [9].

Although the patient initially appeared to fit the criteria for DRESS, her rash was demonstrated to be unrelated to a drug eruption. This left us with the task of determining the cause of her eosinophilia and hepatitis. Several potential causes of hepatitis were considered and tested for, including viral causes of hepatitis, autoimmune disease markers, and iron studies. Ultimately, the presence of immunoglobulin M (IgM) antibodies indicated a primary EBV infection rather than reactivation in the context of DRESS as outlined above.

EBV is ubiquitous in the population and can cause a variety of presentations, from classic infectious mononucleosis to uncommon manifestations such as pneumonia and myocarditis [2]. It frequently presents in childhood and adolescence, and its effects in adulthood are less well known. However, the few available case reports describe EBV-associated hepatitis without the classic symptoms such as sore throat, ranging from mild symptoms of right upper quadrant pain to ascites and pulmonary edema [5]. Even in those severely affected, supportive care was sufficient to allow recovery in two to six months.

As for the elevated eosinophil count, beta-lactam antibiotics may cause asymptomatic peripheral eosinophilia outside the context of DRESS, although much of the literature supporting of moderate quality. A study published in 1999 reviewing the adverse effects of parenteral antibiotics administered outpatient demonstrated that 12% of patients developed asymptomatic peripheral eosinophilia [10]. A more recent look at this question in 2015 determined that 25% of study patients had peripheral eosinophilia, which was primarily associated with the use of vancomycin, penicillin, rifampin, and linezolid. However, 60% of those patients did not have a symptomatic hypersensitivity reaction [11]. Our patient’s eosinophilia therefore may have been associated with her recent amoxicillin use.

## Conclusions

Despite the initial way this case conformed to heuristic associations of DRESS syndrome, it ended up being an unusual presentation of a common disease. Broad diagnostic testing was required to uncover the cause of the patient’s hepatitis, and the cause of her elevated eosinophils was never absolutely determined. Although EBV does not require treatment and this patient recovered with supportive care, other potential causes of hepatitis require a low threshold of suspicion to make the diagnosis and begin treatment early enough to prevent morbidity. This case demonstrates the importance of maintaining a broad differential even in seemingly obvious cases.
